# Starch in Cutaneous Diseases

**Published:** 1852-09

**Authors:** 


*Starch in Cutaneous Diseases.*—M. Cazenave has lately employed, largely, powdered starch, pure or mixed with oxide of zinc or camphor, in various diseases of skin. In acute eczema, impetigo, herpes, acne rosacea, after washing the parts with a weak alkaline solution, and well drying them, some of the following powder is sprinkled, viz: Oxide of zinc, one part; starch powder, fifteen parts. In prurigo of the axillæ, the anus, or the genitals, a quarter part of camphor is added.—*Med. Times & Gazette*, May 22, 1852, from *L'Union Medicale.*

